# Supplementary material for: Benefit take-up in the last year of life: a population-based study using linked data for England and Wales
Source: SSM Popul Health. 2026 Oct 15;36:101986. doi: 10.1016/j.ssmph.2026.101986 (PMC13722064; doi:10.1016/j.ssmph.2026.101986)
Supplement: Supplementary file 1 [file mmc1.docx]

**Supplementary file for:** Benefit take-up in the last year of life: a population-based study using linked data for England and Wales

**Highest level of qualification variable**

Definition of highest level of qualification, categories in the 2011 and 2021 Census data

- No qualifications
- Level 1 and entry level qualifications: 1 to 4 GCSEs grade A* to C, Any GCSEs at other grades, O levels or CSEs (any grades), 1 AS level, NVQ level 1, Foundation GNVQ, Basic or Essential Skills
- Level 2 qualifications: 5 or more GCSEs (A* to C or 9 to 4), O levels (passes), CSEs (grade 1), School Certification, 1 A level, 2 to 3 AS levels, VCEs, Intermediate or Higher Diploma, Welsh Baccalaureate Intermediate Diploma, NVQ level 2, Intermediate GNVQ, City and Guilds Craft, BTEC First or General Diploma, RSA Diploma
- Apprenticeship
- Level 3 qualifications: 2 or more A levels or VCEs, 4 or more AS levels, Higher School Certificate, Progression or Advanced Diploma, Welsh Baccalaureate Advance Diploma, NVQ level 3; Advanced GNVQ, City and Guilds Advanced Craft, ONC, OND, BTEC National, RSA Advanced Diploma
- Level 4 qualifications or above: degree (BA, BSc), higher degree (MA, PhD, PGCE), NVQ level 4 to 5, HNC, HND, RSA Higher Diploma, BTEC Higher level, professional qualifications (for example, teaching, nursing, accountancy)
- Other: vocational or work-related qualifications, other qualifications achieved in England or Wales, qualifications achieved outside England or Wales (equivalent not stated or unknown)

**Table 1: Sample descriptives for all people who died of chronic and sudden* causes of death in England and Wales between 1st May 2018 and 30th April 2021**

|  | **All deaths from sudden causes*** | **Column %** | **All deaths from non-sudden/chronic causes** | **Column %** |  |  |
| --- | --- | --- | --- | --- | --- | --- |
| total | 446067 | 100.0% | 1049493 | 100.0% |  |  |
| Median age (iq range) | 83 (72, 89) |  | 81 (72, 88) |  |  |  |
|  |  |  |  |  |  |  |
| **Working age at death (16-65)** | 72819 | 16.3% | 142432 | 13.6% |  |  |
| Median age (iq range) | 54 (43, 61) |  | 58 (52, 62) |  |  |  |
|  |  |  |  |  |  |  |
| **Pension age at death (≥66)** | 373248 | 83.7% | 907061 | 86.4% |  |  |
| Median age (iq range) | 85 (78, 90) |  | 83 (76, 89) |  |  |  |
| **Sex** |  |  |  |  |  |  |
| Female | 217995 | 48.9% | 528516 | 50.4% |  |  |
| Male | 228072 | 51.1% | 520977 | 49.6% |  |  |
| **Ethnicity** |  |  |  |  |  |  |
| White British | 405463 | 90.9% | 973750 | 92.8% |  |  |
| White other | 13479 | 3.0% | 33337 | 3.2% |  |  |
| Mixed/Multiple ethnicity | 2642 | 0.6% | 4559 | 0.4% |  |  |
| Bangladeshi | 1469 | 0.3% | 1919 | 0.2% |  |  |
| Indian | 6987 | 1.6% | 10822 | 1.0% |  |  |
| Pakistani | 3977 | 0.9% | 5216 | 0.5% |  |  |
| Chinese | 801 | 0.2% | 1575 | 0.2% |  |  |
| Black African | 2155 | 0.5% | 2869 | 0.3% |  |  |
| Black Caribbean | 4673 | 1.0% | 8740 | 0.8% |  |  |
| Other ethnic group | 4161 | 0.9% | 6436 | 0.6% |  |  |
| Missing or not stated | 260 | 0.1% | 270 | 0.0% |  |  |
| **Area-based deprivation** |  |  |  |  |  |  |
| 1 (most deprived) | 92667 | 20.8% | 203890 | 19.4% |  |  |
| 2 | 89534 | 20.1% | 202704 | 19.3% |  |  |
| 3 | 90416 | 20.3% | 214872 | 20.5% |  |  |
| 4 | 88857 | 19.9% | 217052 | 20.7% |  |  |
| 5 (least deprived) | 84181 | 18.9% | 210224 | 20.0% |  |  |
| Missing or not stated | 412 | 0.1% | 751 | 0.1% |  |  |
| **Highest educational level** |  |  |  |  |  |  |
| No qualifications | 234371 | 52.5% | 531903 | 50.7% |  |  |
| Level 1 (1-4 CSEs etc) | 31524 | 7.1% | 76551 | 7.3% |  |  |
| Level 2 (5+ CSEs etc) | 37545 | 8.4% | 90344 | 8.6% |  |  |
| Apprenticeships | 22957 | 5.1% | 59888 | 5.7% |  |  |
| Level 3 (2+ A levels etc) | 20989 | 4.7% | 52099 | 5.0% |  |  |
| Level 4 (degree etc) | 68821 | 15.4% | 176373 | 16.8% |  |  |
| Other qualifications: Vocational/Foreign | 25939 | 5.8% | 61234 | 5.8% |  |  |
| Missing or not stated | 3921 | 0.9% | 1101 | 0.1% |  |  |
| **English/welsh language proficiency** |  |  |  |  |  |  |
| Not well/Not at all | 6388 | 1.4% | 10056 | 1.0% |  |  |
| Very well/Well | 9500 | 2.1% | 15994 | 1.5% |  |  |
| Native English speaker | 429919 | 96.4% | 1023173 | 97.5% |  |  |
| Missing | 260 | 0.1% | 270 | 0.0% |  |  |
| **Region** |  |  |  |  |  |  |
| North East | 24939 | 5.6% | 54322 | 5.2% |  |  |
| North West | 60491 | 13.6% | 143011 | 13.6% |  |  |
| Yorkshire and The Humber | 42066 | 9.4% | 105882 | 10.1% |  |  |
| East Midlands | 36745 | 8.2% | 91325 | 8.7% |  |  |
| West Midlands | 47074 | 10.6% | 109224 | 10.4% |  |  |
| East of England | 47404 | 10.6% | 113338 | 10.8% |  |  |
| London | 46544 | 10.4% | 97069 | 9.2% |  |  |
| South East | 67967 | 15.2% | 159492 | 15.2% |  |  |
| South West | 44857 | 10.1% | 109529 | 10.4% |  |  |
| Wales | 27568 | 6.2% | 65550 | 6.2% |  |  |
| Missing | 412 | 0.1% | 751 | 0.1% |  |  |
| **Underlying cause of death** | - |  |  |  |  |  |
| Cancer | - | 0.0% | 395307 | 37.7% |  |  |
| Dementia/Alzheimer’s/senility | - | 0.0% | 201782 | 19.2% |  |  |
| Respiratory failure | - | 0.0% | 75830 | 7.2% |  |  |
| Heart failure | - | 0.0% | 239402 | 22.8% |  |  |
| Liver disease | - | 0.0% | 21686 | 2.1% |  |  |
| Renal failure | - | 0.0% | 6657 | 0.6% |  |  |
| Neurodegenerative diseases | - | 0.0% | 30063 | 2.9% |  |  |
| HIV | - | 0.0% | 227 | 0.0% |  |  |
| Stroke | - | 0.0% | 78539 | 7.5% |  |  |
| Sudden causes | 335050 | 75.1% | - | 0.0% |  |  |
| Covid | 111017 | 24.9% | - | 0.0% |  |  |
| **Benefit uptake (PIP, AA or DLA)** |  |  |  |  |  |  |
| yes | 272873 | 61.2% | 691669 | 65.9% |  |  |
| no | 173194 | 38.8% | 357824 | 34.1% |  |  |

*Includes all causes of death not included in the main study population (listed in table 2 in the manuscript), e.g. deaths from external injury, sudden, and non-chronic illnesses

**Table 2: The cumulative non-means tested benefit take-up rate per 1,000 deaths over the last year of life, separately for people who died from chronic and sudden* causes, for working age and pension age people**

*Includes all causes of death not included in the main study population (listed in table 2 in the manuscript), e.g. deaths from external injury, sudden, and non-chronic illnesses

**Figure 1: Cumulative take-up rate per 1,000 deaths of non-means tested benefits** (**Personal Independence Payment (PIP), Attendance Allowance (AA) and Disability Living Allowance (DLA)), over the last 12 months of life, for all sudden deaths* in England and Wales between 1st May 2018 and 30th April 2021**

*Includes all causes of death not included in the main study population (listed in table 2 in the manuscript), e.g. deaths from external injury, sudden, and non-chronic illnesses

**Table 3: Description of decedents and take-up of non-means tested disability benefits (Personal Independence Payment (PIP), Attendance Allowance (AA) and Disability Living Allowance (DLA)), by underlying cause of death and year for deaths aged 16 years and over in England and Wales.**

|  | **2018 (1^st^ April to 31^st^ December)** | | | **2019 (1^st^ January to 31^st^ December)** | | | **2020 (1^st^ January to 31^st^ December)** | | | **2021 (1^st^ January to 31^st^ March)** | | |
| --- | --- | --- | --- | --- | --- | --- | --- | --- | --- | --- | --- | --- |
| **Underlying cause of death** | **n in receipt of PIP, AA or DLA in last 12 months of life** | **total decedents** | **percentage of take-up** | **n in receipt of PIP, AA or DLA in last 12 months of life** | **total decedents** | **percentage of take-up** | **n in receipt of PIP, AA or DLA in last 12 months of life** | **total decedents** | **percentage of take-up** | **n in receipt of PIP, AA or DLA in last 12 months of life** | **total decedents** | **percentage of take-up** |
| **HIV** | 24 | 51 | 47.1 | 31 | 74 | 41.9 | 25 | 70 | 35.7 | 13 | 32 | 40.6 |
| **Cancer** | 55520 | 87914 | 63.2 | 82814 | 132171 | 62.7 | 81280 | 132475 | 61.4 | 25420 | 42747 | 59.5 |
| **Dementia** | 35079 | 40913 | 85.7 | 57750 | 67517 | 85.5 | 61238 | 72107 | 84.9 | 17870 | 21245 | 84.1 |
| **Heart failure** | 27377 | 49666 | 55.1 | 43940 | 79782 | 55.1 | 43984 | 81496 | 54.0 | 14788 | 28458 | 52.0 |
| **Liver disease** | 1895 | 4324 | 43.8 | 2969 | 6820 | 43.5 | 3213 | 7795 | 41.2 | 1101 | 2747 | 40.1 |
| **Neurodegenerative** | 5587 | 6219 | 89.8 | 8932 | 9922 | 90.0 | 9461 | 10566 | 89.5 | 2999 | 3356 | 89.4 |
| **Renal failure** | 1093 | 1495 | 73.1 | 1730 | 2380 | 72.7 | 1488 | 2116 | 70.3 | 472 | 666 | 70.9 |
| **Resp failure** | 11678 | 15811 | 73.9 | 20625 | 28202 | 73.1 | 18033 | 24729 | 72.9 | 5073 | 7088 | 71.6 |
| **Stroke** | 10694 | 17004 | 62.9 | 16295 | 26356 | 61.8 | 16023 | 26318 | 60.9 | 5155 | 8861 | 58.2 |
| **Sudden** | 42868 | 70701 | 60.6 | 70816 | 116030 | 61.0 | 66846 | 111691 | 59.9 | 21004 | 36628 | 57.3 |
| **Covid-19** | - | - | - | - | - | - | 45316 | 68988 | 65.7 | 26023 | 42029 | 61.9 |
| **total** | 191815 | 294098 | 65.2 | 305902 | 469254 | 65.2 | 346907 | 538351 | 64.4 | 119918 | 193857 | 61.9 |

**Table 4: Age and sex standardised benefit take-up rate and average deprivation scores for Local Authorities**

| **LA code** | **Local Authority (LA) name** | **Age and sex standardised benefit take-up rate** | **Quintiles of benefit take-up rate (1 is lowest take-up) - for Eng and Wales overall** | **LA average deprivation score (highest is most deprived)** | **Quintiles of LA average deprivation score (1 is most deprived) - derived for Eng and Wales separately*** |
| --- | --- | --- | --- | --- | --- |
| E06000041 | Wokingham | 0.525 | 1 | 5.846 | 5 |
| E09000027 | Richmond upon Thames | 0.535 | 1 | 9.425 | 5 |
| E07000089 | Hart | 0.553 | 1 | 5.544 | 5 |
| E09000020 | Kensington and Chelsea | 0.558 | 1 | 21.526 | 2 |
| E06000037 | West Berkshire | 0.562 | 1 | 9.952 | 5 |
| E06000040 | Windsor and Maidenhead | 0.562 | 1 | 8.376 | 5 |
| E07000084 | Basingstoke and Deane | 0.564 | 1 | 12.823 | 4 |
| E09000032 | Wandsworth | 0.568 | 1 | 16.611 | 3 |
| E06000060 | Buckinghamshire | 0.57 | 1 | 10.074 | 5 |
| E07000207 | Elmbridge | 0.57 | 1 | 7.944 | 5 |
| E07000180 | Vale of White Horse | 0.572 | 1 | 8.358 | 5 |
| E07000094 | Winchester | 0.572 | 1 | 9.615 | 5 |
| E09000033 | Westminster | 0.573 | 1 | 20.339 | 3 |
| E07000085 | East Hampshire | 0.575 | 1 | 10.284 | 5 |
| E07000179 | South Oxfordshire | 0.576 | 1 | 8.459 | 5 |
| E09000021 | Kingston upon Thames | 0.577 | 1 | 11.381 | 5 |
| E09000024 | Merton | 0.58 | 1 | 14.649 | 4 |
| E06000038 | Reading | 0.581 | 1 | 19.619 | 3 |
| E07000068 | Brentwood | 0.581 | 1 | 10.007 | 5 |
| E09000013 | Hammersmith and Fulham | 0.581 | 1 | 22.27 | 2 |
| E07000240 | St Albans | 0.581 | 1 | 8.339 | 5 |
| E09000018 | Hounslow | 0.581 | 1 | 21.487 | 2 |
| E07000102 | Three Rivers | 0.584 | 1 | 9.871 | 5 |
| E07000012 | South Cambridgeshire | 0.585 | 1 | 8.496 | 5 |
| E09000014 | Haringey | 0.588 | 1 | 27.956 | 1 |
| E06000055 | Bedford | 0.588 | 1 | 18.932 | 3 |
| E07000215 | Tandridge | 0.59 | 1 | 11.896 | 5 |
| E06000056 | Central Bedfordshire | 0.59 | 1 | 12.152 | 5 |
| E07000092 | Rushmoor | 0.591 | 1 | 15.903 | 4 |
| E07000087 | Fareham | 0.592 | 1 | 9.019 | 5 |
| E07000111 | Sevenoaks | 0.593 | 1 | 12.437 | 5 |
| E06000036 | Bracknell Forest | 0.593 | 1 | 10.241 | 5 |
| E07000181 | West Oxfordshire | 0.593 | 1 | 8.684 | 5 |
| E07000209 | Guildford | 0.594 | 1 | 9.395 | 5 |
| E07000131 | Harborough | 0.595 | 1 | 8.015 | 5 |
| E07000242 | East Hertfordshire | 0.595 | 1 | 8.188 | 5 |
| E06000017 | Rutland | 0.595 | 1 | 8.381 | 5 |
| E07000212 | Runnymede | 0.596 | 1 | 12.012 | 5 |
| E07000222 | Warwick | 0.597 | 1 | 12.005 | 5 |
| E07000214 | Surrey Heath | 0.598 | 1 | 8.066 | 5 |
| E07000103 | Watford | 0.598 | 1 | 15.41 | 4 |
| E07000078 | Cheltenham | 0.599 | 1 | 14.26 | 4 |
| E07000088 | Gosport | 0.6 | 1 | 20.541 | 3 |
| E07000216 | Waverley | 0.6 | 1 | 7.494 | 5 |
| E07000227 | Horsham | 0.6 | 1 | 9.89 | 5 |
| E09000003 | Barnet | 0.601 | 1 | 16.148 | 4 |
| E09000007 | Camden | 0.601 | 1 | 20.131 | 3 |
| E07000178 | Oxford | 0.602 | 1 | 16.707 | 3 |
| E07000093 | Test Valley | 0.602 | 1 | 11.931 | 5 |
| E07000098 | Hertsmere | 0.602 | 1 | 13.938 | 4 |
| E09000005 | Brent | 0.603 | 1 | 25.558 | 2 |
| E07000008 | Cambridge | 0.604 | 1 | 14.855 | 4 |
| E07000099 | North Hertfordshire | 0.605 | 1 | 11.627 | 5 |
| E07000079 | Cotswold | 0.606 | 1 | 11.061 | 5 |
| E07000211 | Reigate and Banstead | 0.606 | 1 | 11.276 | 5 |
| E09000016 | Havering | 0.606 | 1 | 16.789 | 3 |
| E09000017 | Hillingdon | 0.607 | 1 | 18.223 | 3 |
| E07000082 | Stroud | 0.607 | 1 | 10.797 | 5 |
| E07000133 | Melton | 0.607 | 1 | 12.532 | 4 |
| E07000210 | Mole Valley | 0.607 | 1 | 9.511 | 5 |
| E06000039 | Slough | 0.608 | 1 | 22.965 | 2 |
| E07000091 | New Forest | 0.61 | 1 | 13.015 | 4 |
| E07000177 | Cherwell | 0.611 | 1 | 14.41 | 4 |
| E09000015 | Harrow | 0.611 | 1 | 15.031 | 4 |
| E09000006 | Bromley | 0.611 | 1 | 14.163 | 4 |
| E09000010 | Enfield | 0.611 | 1 | 25.781 | 2 |
| E07000213 | Spelthorne | 0.612 | 2 | 14.943 | 4 |
| E06000022 | Bath and North East Somerset | 0.612 | 2 | 11.745 | 5 |
| E07000072 | Epping Forest | 0.612 | 2 | 15.068 | 4 |
| E07000109 | Gravesham | 0.612 | 2 | 21.414 | 2 |
| E07000044 | South Hams | 0.612 | 2 | 13.724 | 4 |
| E07000187 | Mendip | 0.612 | 2 | 16.6 | 3 |
| E07000116 | Tunbridge Wells | 0.615 | 2 | 11.31 | 5 |
| E07000096 | Dacorum | 0.615 | 2 | 13.004 | 4 |
| E07000070 | Chelmsford | 0.616 | 2 | 12.221 | 5 |
| E07000221 | Stratford-on-Avon | 0.618 | 2 | 11.728 | 5 |
| E07000090 | Havant | 0.618 | 2 | 21.806 | 2 |
| E09000023 | Lewisham | 0.619 | 2 | 26.661 | 2 |
| E07000011 | Huntingdonshire | 0.619 | 2 | 12.55 | 4 |
| E07000225 | Chichester | 0.619 | 2 | 14.085 | 4 |
| E09000029 | Sutton | 0.619 | 2 | 13.987 | 4 |
| E07000147 | North Norfolk | 0.619 | 2 | 21.058 | 3 |
| E07000077 | Uttlesford | 0.62 | 2 | 9.258 | 5 |
| E07000228 | Mid Sussex | 0.62 | 2 | 7.747 | 5 |
| E07000009 | East Cambridgeshire | 0.62 | 2 | 11.507 | 5 |
| E06000062 | West Northamptonshire | 0.62 | 2 | 17.535 | 3 |
| E07000074 | Maldon | 0.621 | 2 | 14.169 | 4 |
| E07000167 | Ryedale | 0.621 | 2 | 15.665 | 4 |
| E07000065 | Wealden | 0.622 | 2 | 12.311 | 5 |
| E09000026 | Redbridge | 0.623 | 2 | 17.203 | 3 |
| E09000011 | Greenwich | 0.624 | 2 | 24.464 | 2 |
| E07000107 | Dartford | 0.624 | 2 | 18.812 | 3 |
| E07000241 | Welwyn Hatfield | 0.625 | 2 | 14.215 | 4 |
| E07000220 | Rugby | 0.626 | 2 | 14.119 | 4 |
| E07000086 | Eastleigh | 0.626 | 2 | 10.192 | 5 |
| E07000140 | South Holland | 0.626 | 2 | 17.896 | 3 |
| E07000110 | Maidstone | 0.626 | 2 | 16.503 | 3 |
| E07000188 | Sedgemoor | 0.627 | 2 | 21.306 | 2 |
| E07000144 | Broadland | 0.627 | 2 | 11.817 | 5 |
| E06000051 | Shropshire | 0.627 | 2 | 17.153 | 3 |
| E09000009 | Ealing | 0.627 | 2 | 22.71 | 2 |
| E07000245 | West Suffolk | 0.627 | 2 | 16.245 | 4 |
| E06000052 | Cornwall and Isles of Scilly | 0.628 | 2 | 23.072 | 2 |
| E06000030 | Swindon | 0.628 | 2 | 18.622 | 3 |
| E07000208 | Epsom and Ewell | 0.628 | 2 | 8.833 | 5 |
| E08000033 | Calderdale | 0.629 | 2 | 26.351 | 2 |
| E07000106 | Canterbury | 0.629 | 2 | 16.798 | 3 |
| E07000115 | Tonbridge and Malling | 0.629 | 2 | 13.333 | 4 |
| E06000044 | Portsmouth | 0.629 | 2 | 26.899 | 1 |
| E07000238 | Wychavon | 0.63 | 2 | 15.766 | 4 |
| E07000105 | Ashford | 0.63 | 2 | 18.546 | 3 |
| E07000067 | Braintree | 0.63 | 2 | 14.723 | 4 |
| E09000002 | Barking and Dagenham | 0.63 | 2 | 32.768 | 1 |
| E07000166 | Richmondshire | 0.63 | 2 | 12.135 | 5 |
| E07000064 | Rother | 0.631 | 2 | 19.768 | 3 |
| E07000132 | Hinckley and Bosworth | 0.631 | 2 | 13.461 | 4 |
| E07000046 | Torridge | 0.631 | 2 | 23.269 | 2 |
| E07000223 | Adur | 0.631 | 2 | 17.594 | 3 |
| E07000229 | Worthing | 0.633 | 2 | 17.012 | 3 |
| E07000164 | Hambleton | 0.633 | 2 | 11.987 | 5 |
| E07000130 | Charnwood | 0.633 | 2 | 13.238 | 4 |
| E07000043 | North Devon | 0.634 | 2 | 20.559 | 3 |
| E07000066 | Basildon | 0.634 | 2 | 23.243 | 2 |
| E07000200 | Babergh | 0.634 | 2 | 14.267 | 4 |
| E09000022 | Lambeth | 0.634 | 2 | 25.422 | 2 |
| E06000054 | Wiltshire | 0.636 | 2 | 13.447 | 4 |
| E07000030 | Eden | 0.636 | 2 | 16.328 | 3 |
| E07000083 | Tewkesbury | 0.636 | 2 | 12.142 | 5 |
| E07000035 | Derbyshire Dales | 0.636 | 2 | 11.895 | 5 |
| E07000196 | South Staffordshire | 0.637 | 2 | 13.124 | 4 |
| E06000025 | South Gloucestershire | 0.638 | 2 | 11.66 | 5 |
| E07000226 | Crawley | 0.639 | 2 | 18.94 | 3 |
| E06000033 | Southend-on-Sea | 0.639 | 2 | 22.375 | 2 |
| E07000224 | Arun | 0.639 | 2 | 18.638 | 3 |
| E09000004 | Bexley | 0.639 | 2 | 16.273 | 4 |
| E07000203 | Mid Suffolk | 0.639 | 2 | 13.225 | 4 |
| E07000176 | Rushcliffe | 0.639 | 2 | 7.18 | 5 |
| E07000047 | West Devon | 0.64 | 3 | 18.052 | 3 |
| E09000031 | Waltham Forest | 0.64 | 3 | 25.209 | 2 |
| E07000172 | Broxtowe | 0.64 | 3 | 14.238 | 4 |
| E07000193 | East Staffordshire | 0.64 | 3 | 19.028 | 3 |
| E06000019 | Herefordshire | 0.64 | 3 | 18.89 | 3 |
| E07000246 | Somerset West and Taunton | 0.64 | 3 | 19.142 | 3 |
| E07000095 | Broxbourne | 0.64 | 3 | 17.989 | 3 |
| E07000189 | South Somerset | 0.64 | 3 | 17.347 | 3 |
| E07000141 | South Kesteven | 0.642 | 3 | 13.499 | 4 |
| E07000113 | Swale | 0.642 | 3 | 27.076 | 1 |
| E06000042 | Milton Keynes | 0.643 | 3 | 17.98 | 3 |
| E07000010 | Fenland | 0.643 | 3 | 25.426 | 2 |
| E06000024 | North Somerset | 0.643 | 3 | 15.825 | 4 |
| E06000046 | Isle of Wight | 0.643 | 3 | 23.294 | 2 |
| E06000059 | Dorset | 0.643 | 3 | 15.735 | 4 |
| E09000008 | Croydon | 0.644 | 3 | 22.477 | 2 |
| E07000148 | Norwich | 0.644 | 3 | 27.599 | 1 |
| E07000244 | East Suffolk | 0.644 | 3 | 19.56 | 3 |
| E06000049 | Cheshire East | 0.645 | 3 | 14.475 | 4 |
| E06000034 | Thurrock | 0.645 | 3 | 20.928 | 3 |
| E07000073 | Harlow | 0.645 | 3 | 21.413 | 2 |
| E07000142 | West Lindsey | 0.645 | 3 | 20.355 | 3 |
| E07000075 | Rochford | 0.645 | 3 | 10.449 | 5 |
| E07000219 | Nuneaton and Bedworth | 0.645 | 3 | 23.54 | 2 |
| E07000217 | Woking | 0.646 | 3 | 10.804 | 5 |
| E07000040 | East Devon | 0.646 | 3 | 12.764 | 4 |
| E07000145 | Great Yarmouth | 0.647 | 3 | 33.097 | 1 |
| E07000173 | Gedling | 0.647 | 3 | 14.894 | 4 |
| E06000032 | Luton | 0.647 | 3 | 25.908 | 2 |
| E07000124 | Ribble Valley | 0.647 | 3 | 10.594 | 5 |
| E07000197 | Stafford | 0.647 | 3 | 13.678 | 4 |
| E07000129 | Blaby | 0.648 | 3 | 10.629 | 5 |
| E07000234 | Bromsgrove | 0.648 | 3 | 11.697 | 5 |
| E07000143 | Breckland | 0.649 | 3 | 19.614 | 3 |
| E07000134 | North West Leicestershire | 0.649 | 3 | 14.573 | 4 |
| E06000058 | Bournemouth, Christchurch and Poole | 0.65 | 3 | 18.173 | 3 |
| E06000026 | Plymouth | 0.65 | 3 | 26.619 | 2 |
| E07000218 | North Warwickshire | 0.65 | 3 | 17.907 | 3 |
| E06000031 | Peterborough | 0.651 | 3 | 27.821 | 1 |
| E07000063 | Lewes | 0.652 | 3 | 15.758 | 4 |
| E07000042 | Mid Devon | 0.652 | 3 | 16.928 | 3 |
| E07000080 | Forest of Dean | 0.652 | 3 | 18.013 | 3 |
| E07000243 | Stevenage | 0.652 | 3 | 19.695 | 3 |
| E06000035 | Medway | 0.653 | 3 | 23.936 | 2 |
| E07000076 | Tendring | 0.654 | 3 | 30.484 | 1 |
| E08000035 | Leeds | 0.654 | 3 | 27.301 | 1 |
| E07000108 | Dover | 0.655 | 3 | 22.161 | 2 |
| E06000061 | North Northamptonshire | 0.655 | 3 | 19.685 | 3 |
| E07000149 | South Norfolk | 0.655 | 3 | 13.318 | 4 |
| E07000239 | Wyre Forest | 0.656 | 3 | 22.437 | 2 |
| E08000029 | Solihull | 0.656 | 3 | 17.37 | 3 |
| E07000119 | Fylde | 0.656 | 3 | 15.875 | 4 |
| E07000175 | Newark and Sherwood | 0.657 | 3 | 19.227 | 3 |
| E07000081 | Gloucester | 0.657 | 3 | 21.807 | 2 |
| E06000005 | Darlington | 0.657 | 3 | 25.657 | 2 |
| E07000139 | North Kesteven | 0.657 | 3 | 11.553 | 5 |
| E07000199 | Tamworth | 0.658 | 3 | 21.061 | 3 |
| E07000045 | Teignbridge | 0.658 | 3 | 15.893 | 4 |
| E06000045 | Southampton | 0.658 | 3 | 26.876 | 1 |
| E09000019 | Islington | 0.658 | 3 | 27.535 | 1 |
| E09000028 | Southwark | 0.659 | 3 | 25.811 | 2 |
| E07000069 | Castle Point | 0.659 | 3 | 16.842 | 3 |
| E07000198 | Staffordshire Moorlands | 0.66 | 4 | 15.04 | 4 |
| E06000043 | Brighton and Hove | 0.66 | 4 | 20.761 | 3 |
| E07000039 | South Derbyshire | 0.66 | 4 | 14.494 | 4 |
| E06000011 | East Riding of Yorkshire | 0.661 | 4 | 15.605 | 4 |
| E07000041 | Exeter | 0.661 | 4 | 16.215 | 4 |
| E07000194 | Lichfield | 0.661 | 4 | 12.566 | 4 |
| E06000018 | Nottingham | 0.661 | 4 | 34.891 | 1 |
| E06000015 | Derby | 0.661 | 4 | 26.323 | 2 |
| E09000012 | Hackney and City of London | 0.662 | 4 | 32.526 | 1 |
| E07000114 | Thanet | 0.662 | 4 | 31.314 | 1 |
| E07000235 | Malvern Hills | 0.662 | 4 | 16.066 | 4 |
| E07000112 | Folkestone and Hythe | 0.662 | 4 | 24.149 | 2 |
| W06000009 | Pembrokeshire | 0.663 | 4 | 19.61966 | 4 |
| E08000034 | Kirklees | 0.663 | 4 | 25.151 | 2 |
| E06000014 | York | 0.664 | 4 | 11.727 | 5 |
| E07000026 | Allerdale | 0.664 | 4 | 22.938 | 2 |
| E08000009 | Trafford | 0.666 | 4 | 16.088 | 4 |
| E09000025 | Newham | 0.667 | 4 | 29.577 | 1 |
| E08000027 | Dudley | 0.668 | 4 | 24.103 | 2 |
| W06000001 | Isle of Anglesey | 0.668 | 4 | 18.27421 | 4 |
| E06000016 | Leicester | 0.669 | 4 | 30.877 | 1 |
| E07000118 | Chorley | 0.669 | 4 | 16.863 | 3 |
| E09000030 | Tower Hamlets | 0.67 | 4 | 27.913 | 1 |
| E06000007 | Warrington | 0.671 | 4 | 18.942 | 3 |
| E06000050 | Cheshire West and Chester | 0.671 | 4 | 18.083 | 3 |
| E07000169 | Selby | 0.671 | 4 | 12.73 | 4 |
| E06000023 | Bristol | 0.672 | 4 | 26.363 | 2 |
| W06000005 | Flintshire | 0.672 | 4 | 15.47085 | 5 |
| E07000237 | Worcester | 0.673 | 4 | 20.414 | 3 |
| E08000032 | Bradford | 0.673 | 4 | 34.666 | 1 |
| E07000136 | Boston | 0.673 | 4 | 22.967 | 2 |
| E07000165 | Harrogate | 0.673 | 4 | 10.897 | 5 |
| E07000236 | Redditch | 0.674 | 4 | 22.524 | 2 |
| E07000062 | Hastings | 0.674 | 4 | 34.281 | 1 |
| E07000028 | Carlisle | 0.675 | 4 | 21.997 | 2 |
| E08000026 | Coventry | 0.675 | 4 | 25.613 | 2 |
| E08000008 | Tameside | 0.675 | 4 | 31.374 | 1 |
| E07000125 | Rossendale | 0.675 | 4 | 24.062 | 2 |
| E07000138 | Lincoln | 0.675 | 4 | 26.889 | 1 |
| E07000146 | King's Lynn and West Norfolk | 0.675 | 4 | 23.72 | 2 |
| E06000057 | Northumberland | 0.675 | 4 | 22.079 | 2 |
| E07000071 | Colchester | 0.676 | 4 | 16.778 | 3 |
| W06000023 | Powys | 0.678 | 4 | 15.75096 | 5 |
| E08000022 | North Tyneside | 0.679 | 4 | 22.279 | 2 |
| E08000005 | Rochdale | 0.68 | 4 | 34.415 | 1 |
| E07000163 | Craven | 0.681 | 4 | 12.76 | 4 |
| E06000012 | North East Lincolnshire | 0.681 | 4 | 31.335 | 1 |
| E07000061 | Eastbourne | 0.682 | 4 | 22.11 | 2 |
| E07000137 | East Lindsey | 0.682 | 4 | 29.892 | 1 |
| W06000008 | Ceredigion | 0.682 | 4 | 17.13084 | 4 |
| E07000126 | South Ribble | 0.683 | 4 | 15.33 | 4 |
| E08000004 | Oldham | 0.683 | 4 | 33.155 | 1 |
| W06000002 | Gwynedd | 0.683 | 4 | 16.85932 | 5 |
| E06000003 | Redcar and Cleveland | 0.684 | 4 | 29.792 | 1 |
| E08000002 | Bury | 0.685 | 4 | 23.682 | 2 |
| E07000036 | Erewash | 0.689 | 4 | 18.818 | 3 |
| E07000135 | Oadby and Wigston | 0.689 | 4 | 12.958 | 4 |
| E06000013 | North Lincolnshire | 0.689 | 4 | 22.096 | 2 |
| E07000120 | Hyndburn | 0.689 | 4 | 34.333 | 1 |
| E06000002 | Middlesbrough | 0.689 | 4 | 40.46 | 1 |
| E06000008 | Blackburn with Darwen | 0.689 | 4 | 36.013 | 1 |
| E07000122 | Pendle | 0.69 | 4 | 30.723 | 1 |
| W06000021 | Monmouthshire | 0.69 | 4 | 13.25528 | 5 |
| E07000031 | South Lakeland | 0.69 | 4 | 12.501 | 4 |
| E07000202 | Ipswich | 0.691 | 4 | 25.89 | 2 |
| E06000027 | Torbay | 0.691 | 4 | 28.104 | 1 |
| E08000007 | Stockport | 0.692 | 5 | 20.826 | 3 |
| E07000168 | Scarborough | 0.692 | 5 | 26.28 | 2 |
| E07000127 | West Lancashire | 0.692 | 5 | 18.645 | 3 |
| E07000128 | Wyre | 0.693 | 5 | 20.858 | 3 |
| E08000031 | Wolverhampton | 0.694 | 5 | 32.102 | 1 |
| E07000123 | Preston | 0.695 | 5 | 29.531 | 1 |
| E08000025 | Birmingham | 0.697 | 5 | 38.067 | 1 |
| E07000032 | Amber Valley | 0.697 | 5 | 17.97 | 3 |
| E07000192 | Cannock Chase | 0.697 | 5 | 20.426 | 3 |
| E08000019 | Sheffield | 0.7 | 5 | 27.06 | 1 |
| E08000030 | Walsall | 0.701 | 5 | 31.555 | 1 |
| E07000121 | Lancaster | 0.701 | 5 | 24.165 | 2 |
| E07000037 | High Peak | 0.701 | 5 | 15.642 | 4 |
| E06000021 | Stoke-on-Trent | 0.702 | 5 | 34.504 | 1 |
| W06000014 | Vale of Glamorgan | 0.705 | 5 | 14.7174 | 5 |
| E08000014 | Sefton | 0.705 | 5 | 27.035 | 1 |
| E08000001 | Bolton | 0.705 | 5 | 30.691 | 1 |
| W06000010 | Carmarthenshire | 0.706 | 5 | 20.94517 | 3 |
| E07000170 | Ashfield | 0.706 | 5 | 26.308 | 2 |
| E06000010 | Kingston upon Hull | 0.706 | 5 | 40.564 | 1 |
| E08000023 | South Tyneside | 0.706 | 5 | 31.509 | 1 |
| E07000171 | Bassetlaw | 0.706 | 5 | 22.588 | 2 |
| E08000017 | Doncaster | 0.708 | 5 | 30.289 | 1 |
| E06000020 | Telford and Wrekin | 0.709 | 5 | 24.988 | 2 |
| E06000004 | Stockton-on-Tees | 0.71 | 5 | 25.79 | 2 |
| E08000010 | Wigan | 0.71 | 5 | 25.713 | 2 |
| E08000037 | Gateshead | 0.711 | 5 | 28.217 | 1 |
| W06000003 | Conwy | 0.712 | 5 | 18.29115 | 4 |
| E07000174 | Mansfield | 0.713 | 5 | 28.503 | 1 |
| E08000016 | Barnsley | 0.713 | 5 | 29.933 | 1 |
| W06000004 | Denbighshire | 0.714 | 5 | 22.88296 | 3 |
| E07000117 | Burnley | 0.714 | 5 | 37.793 | 1 |
| E08000028 | Sandwell | 0.714 | 5 | 34.884 | 1 |
| E07000195 | Newcastle-under-Lyme | 0.715 | 5 | 18.929 | 3 |
| E07000038 | North East Derbyshire | 0.717 | 5 | 17.399 | 3 |
| W06000006 | Wrexham | 0.717 | 5 | 19.73184 | 3 |
| E08000015 | Wirral | 0.718 | 5 | 29.589 | 1 |
| E06000047 | County Durham | 0.719 | 5 | 26.793 | 2 |
| E08000006 | Salford | 0.719 | 5 | 34.21 | 1 |
| E06000009 | Blackpool | 0.72 | 5 | 45.039 | 1 |
| E08000003 | Manchester | 0.72 | 5 | 40.005 | 1 |
| E07000029 | Copeland | 0.721 | 5 | 25.012 | 2 |
| W06000022 | Newport | 0.73 | 5 | 26.87337 | 1 |
| W06000011 | Swansea | 0.73 | 5 | 21.82918 | 3 |
| E08000036 | Wakefield | 0.732 | 5 | 27.306 | 1 |
| W06000015 | Cardiff | 0.733 | 5 | 23.05759 | 2 |
| E08000021 | Newcastle upon Tyne | 0.737 | 5 | 29.79 | 1 |
| E08000024 | Sunderland | 0.74 | 5 | 30.586 | 1 |
| E07000034 | Chesterfield | 0.74 | 5 | 24.926 | 2 |
| E08000013 | St. Helens | 0.741 | 5 | 31.518 | 1 |
| E08000012 | Liverpool | 0.745 | 5 | 42.412 | 1 |
| E07000027 | Barrow-in-Furness | 0.746 | 5 | 31.117 | 1 |
| W06000020 | Torfaen | 0.746 | 5 | 23.58898 | 2 |
| W06000013 | Bridgend | 0.752 | 5 | 21.18083 | 3 |
| E06000006 | Halton | 0.752 | 5 | 32.325 | 1 |
| E08000018 | Rotherham | 0.754 | 5 | 29.55 | 1 |
| E07000033 | Bolsover | 0.756 | 5 | 25.047 | 2 |
| E06000001 | Hartlepool | 0.757 | 5 | 35.037 | 1 |
| W06000012 | Neath Port Talbot | 0.762 | 5 | 27.37671 | 1 |
| W06000016 | Rhondda Cynon Taf | 0.767 | 5 | 26.62592 | 2 |
| W06000018 | Caerphilly | 0.774 | 5 | 24.57687 | 2 |
| E08000011 | Knowsley | 0.779 | 5 | 43.006 | 1 |
| W06000019 | Blaenau Gwent | 0.78 | 5 | 31.80486 | 1 |
| W06000024 | Merthyr Tydfil | 0.783 | 5 | 29.47156 | 1 |

*The Welsh Index of Multiple Deprivation (WIMD) and the Index of Multiple Deprivation for England (IMD) are separate indices with similar indicators but derived separately for each nation. Scores should not be compared across nations. We have derived the quintiles separately for each nation and note that the quintiles (and the scores) are not comparable between the two nations.

**Figure 2: Relationship between age (modelled using natural spline) and benefit take-up (presented using estimated marginal means for each year of age), adjusted for sex***

*IRR (95% CI) from Poisson model, regressing benefit take-up on age (spline) and sex – NB these estimates are from the ‘minimally adjusted model’ (corresponding to the models reported in table 3 in the manuscript):

Age 16-75: 1.21 [1.2-1.22]

Age 76-85: 1.38 [1.37-1.39]

Age 86-100+: [1.33-1.34]

NB: In figure 2, due to small counts, people aged 20 and under are grouped, and people aged 100 and over are grouped. This grouping is not used in the modelling reported in the main analysis.

**Interaction effects and marginal predicted proportions, testing binary age (working versus pension age) as a moderator of the other exposure effects (age spline as previously described in included)**

NB predicted proportions for each group are calculated at the mean age of each group (56 years old for people of working age, and 83 years old for people of pension age). Models are adjusted for age spline and sex.

**Table 5.a age#ethnicity**

|  | **estimate** | **Pr(>\|z\|)** | **working age, marginal proportion (95%CI)** | **pension age, marginal proportion (95% CI)** |
| --- | --- | --- | --- | --- |
| White British | ref | ref | 0.56 [0.55-0.56] | 0.67 [0.66-0.67] |
| Bangladeshi | 0.15 | 0.00 | 0.58 [0.52-0.64] | 0.81 [0.76-0.86] |
| Black African | 0.18 | 0.00 | 0.45 [0.42-0.49] | 0.65 [0.61-0.69] |
| Black Caribbean | 0.07 | 0.00 | 0.54 [0.51-0.58] | 0.69 [0.67-0.71] |
| Chinese | 0.03 | 0.60 | 0.44 [0.38-0.51] | 0.54 [0.5-0.59] |
| Indian | 0.18 | 0.00 | 0.47 [0.44-0.5] | 0.67 [0.65-0.69] |
| Mixed | -0.02 | 0.47 | 0.59 [0.55-0.63] | 0.69 [0.66-0.72] |
| Other | 0.07 | 0.01 | 0.52 [0.49-0.55] | 0.67 [0.64-0.69] |
| Pakistani | 0.14 | 0.00 | 0.55 [0.52-0.59] | 0.77 [0.74-0.8] |
| White other | 0.07 | 0.00 | 0.51 [0.49-0.53] | 0.66 [0.65-0.66] |

**Fig 4.a age#ethnicity**

**Table 5.b: age#deprivation**

| Area-based deprivation | **estimate** | **Pr(>\|z\|)** | **working age, marginal proportion (95%CI)** | **pension age, marginal proportion (95% CI)** |
| --- | --- | --- | --- | --- |
| 1 (most deprived) | -0.11 | 0.00 | 0.62 [0.61-0.63] | 0.73 [0.73-0.74] |
| 2 | -0.09 | 0.00 | 0.57 [0.56-0.58] | 0.68 [0.68-0.69] |
| 3 | -0.06 | 0.00 | 0.53 [0.52-0.54] | 0.66 [0.66-0.66] |
| 4 | -0.04 | 0.00 | 0.51 [0.5-0.52] | 0.64 [0.64-0.65] |
| 5 (least deprived) | ref | ref | 0.47 [0.46-0.48] | 0.62 [0.62-0.63] |

**Fig 4.b: age#deprivation**

**Table 5.c: age#region**

| Region | **estimate** | **Pr(>\|z\|)** | **working age, marginal proportion (95%CI)** | **pension age, marginal proportion (95% CI)** |
| --- | --- | --- | --- | --- |
| London | ref | ref | 0.52 [0.51-0.53] | 0.62 [0.61-0.62] |
| East Midlands | 0.05 | 0.00 | 0.54 [0.52-0.55] | 0.67 [0.67-0.68] |
| East of England | 0.03 | 0.01 | 0.52 [0.51-0.53] | 0.64 [0.63-0.64] |
| North East | -0.01 | 0.24 | 0.61 [0.59-0.63] | 0.72 [0.71-0.72] |
| North West | 0.01 | 0.43 | 0.59 [0.58-0.6] | 0.71 [0.7-0.71] |
| South East | 0.01 | 0.20 | 0.51 [0.5-0.52] | 0.62 [0.61-0.62] |
| South West | -0.01 | 0.29 | 0.55 [0.53-0.56] | 0.64 [0.64-0.65] |
| Wales | 0.03 | 0.01 | 0.59 [0.58-0.61] | 0.73 [0.72-0.74] |
| West Midlands | 0.07 | 0.00 | 0.53 [0.52-0.55] | 0.68 [0.67-0.69] |
| Yorkshire and The Humber | 0.00 | 0.96 | 0.58 [0.57-0.59] | 0.69 [0.68-0.69] |

**Fig 4.c: age#region**

**Table 5.d: age#underlying cause of death**

| Underlying cause of death | **estimate** | **Pr(>\|z\|)** | **working age, marginal proportion (95%CI)** | **pension age, marginal proportion (95% CI)** |
| --- | --- | --- | --- | --- |
| cancer | ref | ref | 0.63 [0.62-0.63] | 0.63 [0.63-0.64] |
| dementia | -0.04 | 0.00 | 0.81 [0.77-0.86] | 0.79 [0.78-0.79] |
| heart_failure | 0.40 | 0.00 | 0.37 [0.36-0.38] | 0.56 [0.56-0.56] |
| HIV | 0.20 | 0.26 | 0.41 [0.32-0.52] | 0.5 [0.34-0.73] |
| liver_disease | 0.33 | 0.00 | 0.38 [0.37-0.39] | 0.53 [0.51-0.55] |
| neurodegenerative | -0.02 | 0.01 | 0.92 [0.89-0.95] | 0.91 [0.9-0.92] |
| renal_failure | -0.10 | 0.00 | 0.74 [0.65-0.84] | 0.67 [0.65-0.69] |
| resp_failure | -0.01 | 0.20 | 0.74 [0.72-0.76] | 0.74 [0.73-0.74] |
| stroke | 0.50 | 0.00 | 0.37 [0.35-0.38] | 0.61 [0.61-0.62] |

**Fig 4.d: age#underlying cause of death**

**Table 5.e: age#highest educational qualification**

| Highest educational qualification | **estimate** | **Pr(>\|z\|)** | **working age, marginal proportion (95%CI)** | **pension age, marginal proportion (95% CI)** |
| --- | --- | --- | --- | --- |
| no qualifications | ref | ref | 0.66 [0.65-0.66] | 0.69 [0.69-0.7] |
| Apprenticeships | 0.20 | 0.00 | 0.52 [0.5-0.54] | 0.67 [0.66-0.67] |
| Level 1 | 0.11 | 0.00 | 0.54 [0.53-0.55] | 0.64 [0.63-0.64] |
| Level 2 | 0.10 | 0.00 | 0.53 [0.52-0.54] | 0.62 [0.62-0.63] |
| Level 3 | 0.14 | 0.00 | 0.51 [0.5-0.52] | 0.63 [0.62-0.63] |
| Level 4 | 0.19 | 0.00 | 0.47 [0.46-0.48] | 0.6 [0.6-0.61] |
| Other: Vocational/Foreign | 0.16 | 0.00 | 0.54 [0.52-0.56] | 0.67 [0.66-0.68] |

**Fig 4.e: age#highest educational qualification**

**Table 5.f: age#language proficiency**

| English/Welsh language proficiency | **estimate** | **Pr(>\|z\|)** | **working age, marginal proportion (95%CI)** | **pension age, marginal proportion (95% CI)** |
| --- | --- | --- | --- | --- |
| very well/native speaker | ref | ref | 0.55 [0.55-0.55] | 0.67 [0.66-0.67] |
| Not well/not at all | 0.09 | 0.00 | 0.56 [0.53-0.60] | 0.74 [0.72-0.76] |

**Fig 4.f: age#language proficiency**
